# Fibronectin 1 mRNA expression correlates with advanced disease in renal cancer

**DOI:** 10.1186/1471-2407-10-503

**Published:** 2010-09-22

**Authors:** Sandra Waalkes, Faranaz Atschekzei, Mario W Kramer, Jörg Hennenlotter, Gesa Vetter, Jan U Becker, Arnulf Stenzl, Axel S Merseburger, Andres J Schrader, Markus A Kuczyk, Jürgen Serth

**Affiliations:** 1 Department of Urology, Hannover Medical School, Germany; 2 Department of Urology, Eberhard Karls University Tuebingen, Germany; 3 Department of Urology, Ulm University Medical School, Ulm, Germany

## Abstract

**Background:**

Fibronectin 1 (*FN1*) is a glycoprotein involved in cellular adhesion and migration processes. The aim of this study was to elucidate the role of *FN1 *in development of renal cell cancer (RCC) and to determine a prognostic relevance for optimal clinical management.

**Methods:**

212 renal tissue samples (109 RCC, 86 corresponding tissues from adjacent normal renal tissue and 17 oncocytomas) were collected from patients undergoing surgery for renal tumors and subjected to total RNA extraction. Detection of *FN1 *mRNA expression was performed using quantitative real time PCR, three endogenous controls, renal proximal tubular epithelial cells (RPTEC) as biological control and the ΔΔCt method for calculation of relative quantities.

**Results:**

Mean tissue specific *FN1 *mRNA expression was found to be increased approximately seven fold comparing RCC and corresponding kidney control tissues (p < 0.001; ANOVA). Furthermore, tissue specific mean *FN1 *expression was increased approx. 11 fold in clear cell compared to papillary RCC (p = 9×10^-5^; Wilcoxon rank sum test). Patients with advanced disease had higher *FN1 *expression when compared to organ-confined disease (p < 0.001; Wilcoxon rank sum test). Applying subgroup analysis we found a significantly higher *FN1 *mRNA expression between organ-confined and advanced disease in the papillary and not in the clear cell RCC group (p = 0.02 vs. p = 0.2; Wilcoxon rank sum test). There was an increased expression in RCC compared to oncocytoma (p = 0.016; ANOVA).

**Conclusions:**

To our knowledge, this is the first study to show that *FN1 *mRNA expression is higher in RCC compared to normal renal tissue. *FN1 *mRNA expression might serve as a marker for RCC aggressiveness, indicating early systemic progression particularly for patients with papillary RCC.

## Background

Renal cell carcinoma (RCC) is a common urologic tumor and accounts for about 3% of all human malignancies [1]. The annual mortality-to-incidence ratio of RCC is significantly higher compared to other urological malignancies, and its incidence has been increasing steadily in recent decades [2]. Interestingly, even organ-confined RCC of comparable stage and grade can demonstrate a significantly-varying tendency towards tumor progression and systemic spread. Correspondingly, the development of metastases can be observed in a substantial number of patients with tumors initially classified as stage T1b or T2 even more than five years after the initial treatment, hereby demonstrating the limited value of classical patients' and tumor characteristics such as tumor stage and grade to predict the clinical outcome of an individual patient.

An essential step in local disease progression and in the formation of metastases is the invasion of tumour cells into the extracellular matrix. Cell adhesion molecules and extra-cellular matrix proteins support either an increase or a decrease in the ability of tumours cells to adhere to surrounding tissue. Among the extracellular matrix proteins identified, *Fibronectin *(*FN*) seems to play an important role in both inhibition and promotion of cellular attachment by interacting with different receptors.

*FN *is a glycoprotein that is involved in cellular adhesion and migration processes including embryogenesis, wound healing, blood coagulation, host defense, and metastasis. The molecule is widely distributed in healthy membrane, in the lamina propria, in vessel structures, nerves and smooth-muscle cell layers [3]. However, up to now the function of *FN *is not clearly known [4,5]. Few studies described the potential role of *FN *in different malignancies [5-8]. For example in hepatocellular carcinoma an overexpression of *FN *protein was found [7]. Elevated plasma levels were detected in patients suffering from gastrointestinal and head/neck cancer [5]. In non-malignant diseases particularly in thrombosis, hemostasis, vascular disease and platelet function the definitive role of *FN *is still unclear [9-11].

The aim of this study is to elucidate a possible role of *FN1 *in the development of RCC using mRNA expression analyses. 212 renal tissue samples from patients undergoing surgery for renal tumors were analysed using quantitative real time PCR. *FN1 *mRNA expression was significantly increased in RCC compared to corresponding normal renal tissue. Furthermore, our data suggest *FN1 *as marker for progressive disease especially in papillary RCC.

## Methods

### Tissue specimens

Tumors and corresponding tumour free tissue of 126 patients subjected to kidney surgery between 2001 and 2005 collected from the Eberhard Karls University Tuebingen were included in the present investigation. All tumors were freshly obtained from the urological department's operation room. The ethical committee of the institution approved the study. Two pathologists evaluated all specimens with respect to tumor stage, grade and histological subtypes. Tumor stages were assessed according to the UICC 2002 issue of the TNM system [12] and nuclear grading was based on the Fuhrman grading system [13]. Histological subtypes were assessed according to the consensus classification of renal cell neoplasia [14] Organ-confined RCC was defined as pT ≤ 2 and N0/M0 and advanced as pT≥3 and/or N+/M+. The resected tissues were stored at -80°C. Data were collected by physicians and data managers and subsequently maintained by a relational database. Clinical and histopathological data are summarized in table 1.

**Table 1:** Clinical and histopathological data of patients with renal cell cancer

Clinico-pathological parameters	Number of patients	%
Total	109	100

Age (mean; ± SD)	63 ± 11.9	

male	70	64.2

female	39	35.8

Side		

left	49	45

right	60	55

Surgery		

PN	27	24.8

RN	82	75.2

Histology		

clear cell	78	71.6

papillary	22	20.2

chromophobe	2	1.8

other/not classified	7	6.4

Stage		

pT1a	34	31.2

pT1b	30	27.5

pT2	5	4.6

pT3a	13	11.9

pT3b/c	27	24.8

pT4	0	0

LN metastasis ^1^	11	10.1

Pulmonal/visceral metastasis ^1^	23	21.1

Advanced/metastatic disease (pT3-4 and/or N/M+)	48	44.0

Grade		

G1	18	16.5

G1-2	15	13.8

G2	58	53.2

G2-3	7	6.4

G3	11	10.1

^1 ^at time of renal surgery;

Abbreviations: SD = standard deviation, PN = partial nephrectomy, RN = radical nephrectomy, LN = lymph node.

### Primary cells

Renal proximal tubular epithelial cells (RPTEC) were obtained from Lonza (Basel, Switzerland) and cultured according to the manufactures recommendations.

### Quantitative real-time PCR analysis

Total RNA was isolated from 20 cyro sections (each 20 μm) using TriReagent (Ambion) according to the manufacturers' instructions. Furthermore, of each tissue sample two sections were stained with hematoxylin-eosin and evaluated by a pathologist (J.U.B). Total RNA was reverse-transcribed into single-strand complementary DNA (cDNA) using the High Capacity cDNA Reverse Transcription Kit (Applied Biosystems, Foster City, CA, USA). Quantitative real time PCR analyses were performed in duplicate with an ABI 7900 Fast Sequence Detection System using TaqMan gene expression assays and universal PCR master mix according to the manufacturer's specifications (Applied Biosystems). The TaqMan assays were *FN1 *(Assay ID: Hs00365058_m1), *GUSB *(Hs00939627_m1), *RPL13A *(Hs03043885_g1) and *HPRT1 *(Hs99999909_m1). The human *GUSB*, *RPL13A *and *HPRT1 *transcripts served as endogenous controls; cDNA derived from RPTEC primary cell transcripts served as biological control. Additional no-template, no reverse transcription and blank controls were included in each run.

Data were evaluated using the SDS 2.3 Manager, dataAssist V1.0 software and the ΔΔCt method [15,16]. The three endogenous controls were combined using the dataAssist software V1.0 and "arithmetic mean" as normalization method. The method of Livak et al [16] and reference ΔCt values obtained from the biological control RPTEC were applied for calculation of ΔΔCt and relative quantity values, respectively. All statistics were done using the R-package 2.10.0 and Java Gui for R 1.7-0. For visual comparison of univariate data we used bean plots as a combination of one-dimensional scatter plots and density plots were generated using the bean plot package for R statistical software [17]. In all tests, p < 0.05 was considered to indicate significance.

## Results

### *FN1 *mRNA expression in normal renal tissues, RCC and oncocytomas

**Figure 1:**
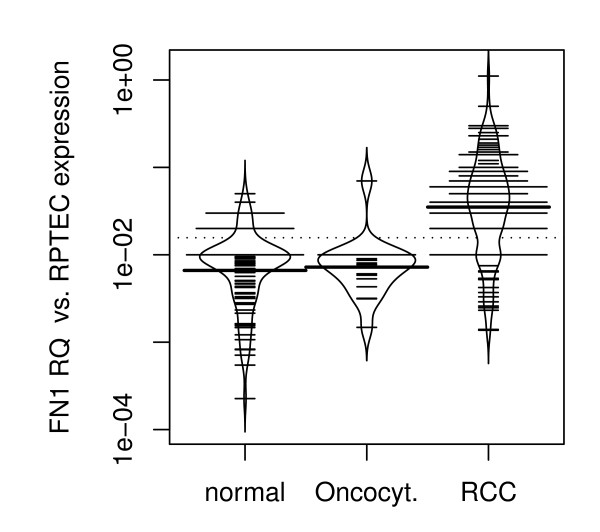
**Bean plot analysis of relative *FN1 *mRNA expression in normal renal tissue, oncocytoma and RCC**. Bean plots represent an alternative to boxplots. The individual observations are shown as small lines in a one-dimensional scatter plot, the estimated density of the distributions is visible and the average is shown. Mean tissue specific *FN1 *mRNA expression was increased approx. 7 fold in RCC compared to corresponding normal renal tissue (p < 0.001) and oncocytoma (p = 0.016). There was no significant difference in FN1 expression between normal renal tissue and oncocytoma. Abbreviations: FN1RQ = *Relative quantity of FN1 mRNA expression analysis using RPTEC primary cells as a biological calibrator*; normal = *normal renal tissue; *Oncocyt = *oncocytoma*.

In order to examine the mRNA expression of *FN1 *in RCC, 109 tumour RNA samples and corresponding histopathological normal renal tissue of 86 of these patients were analysed using quantitative real time PCR. Furthermore, 17 tissue samples of oncocytomas were examined. Expression of *FN1 *was observed in all analysed samples. Mean tissue specific *FN1 *mRNA expression was found to be increased approximately seven fold comparing RCC and corresponding kidney control tissues (p < 0.001; ANOVA). There was no significant difference in *FN1 *expression between normal renal tissue and oncocytoma. However, there was an increased expression in RCC compared to oncocytoma (p = 0.016; ANOVA; figure 1, table 2).

**Table 2:** *FN1 *mRNA expression in normal renal tissue, RCC and oncocytoma

Tissue	Number of tissues	FN1RQ
Normal	86	0.0118

RCC	109	0.0821

Oncocytoma	17	0.0117

Total	212	

Abbreviations: FN1RQ = *Relative quantity of FN1 mRNA expression*.

### Clinical observations

We investigated if any correlations of interest could be seen between the expression of *FN1 *and clinical parameters (table 1). The mean age of the cohort was 63 years (SD ± 11.9). Seventy patients were men (64.2%) and 39 patients were women (35.8%). Seventy-eight patients presented with clear cell RCC, 22 patients with papillary RCCs, and two patients with chromophobe RCC and seven patients with unclassified histology.

**Figure 2:**
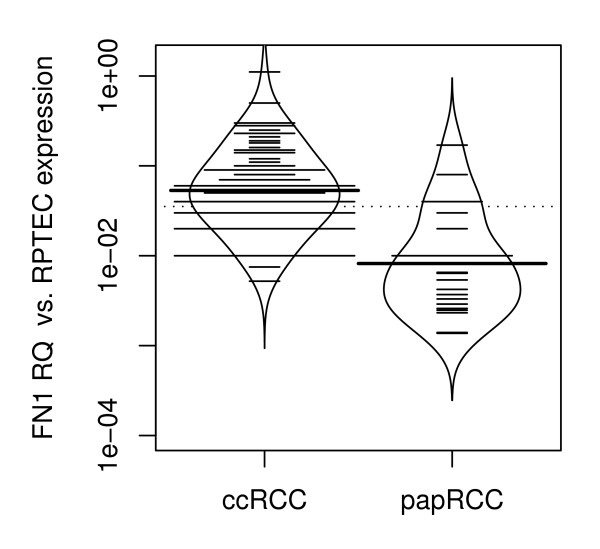
***FN1 *mRNA expression in clear cell RCC and papillary RCC**. Mean tissue specific *FN1 *expression was increased approx. 11 fold in clear cell RCC compared to papillary RCC (p < 0.001). Abbreviations: FN1RQ = *Relative quantity of FN1 mRNA expression analysis using RPTEC primary cells as a biological calibrator; *ccRCC = *clear cell RCC; *pRCC = *papillary RCC*.

We found a significant difference in *FN1 *mRNA expression between clear cell RCC and papillary RCC. The tissue specific mean *FN1 *expression was increased approx. 11 fold in clear cell RCC compared to papillary RCC (p = 9 ×10^-5^; Wilcoxon rank sum test; figure 2). No significant correlation was observed for histological grading, TNM group stage, lymph node metastasis and the presence of distant metastases (Spearman R nonparametric correlation analysis).

**Figure 3:**
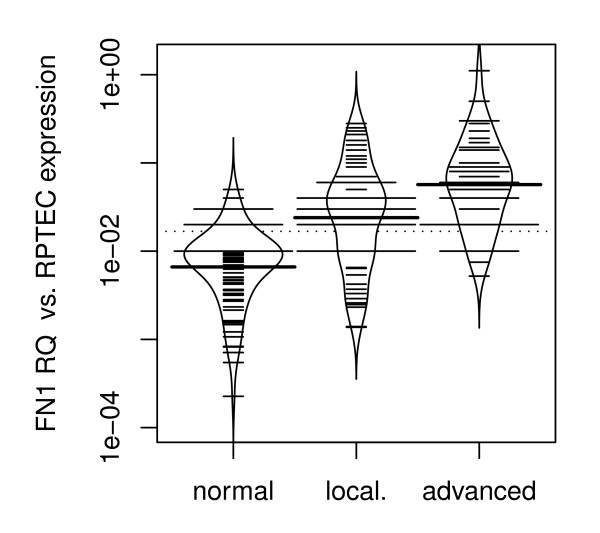
**Bean plot analysis of stage specific Expression: *FN1 *mRNA expression in organ-confined (pT ≤ 2 and N0/M0) and advanced RCC (pT≥3 and/or N+/M+), with a higher *FN1 *expression in advanced disease (p < 0.001)**. Abbreviations: FN1RQ = *Relative quantity of FN1 mRNA expression analysis using RPTEC primary cells as a biological calibrator; *normal = *normal renal tissue; *local = *organ-confined RCC; *advanced = *advanced RCC*.

**Figure 4:**
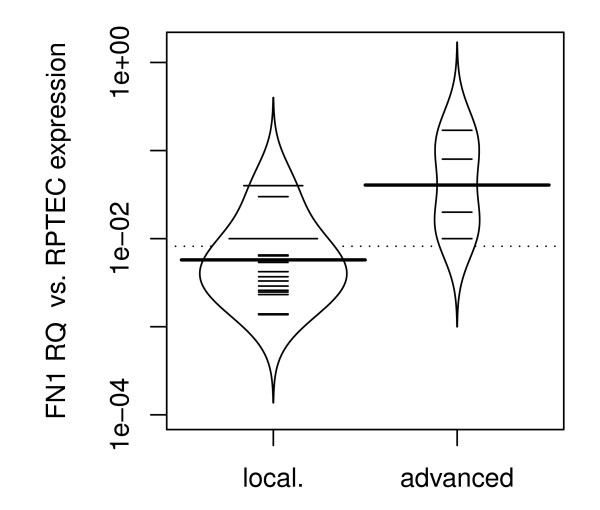
**Progression specific Expression in papillary RCC: higher *FN1 *mRNA in advanced (pT≥3 and/or N+/M+) compared to organ-confined (pT ≤ 2 and N0/M0) RCC (p = 0.017)**. Abbreviations: FN1RQ = *Relative quantity of FN1 mRNA expression analysis using RPTEC primary cells as a biological calibrator; *local = *organ-confined RCC; *advanced = *advanced RCC*.

**Figure 5:**
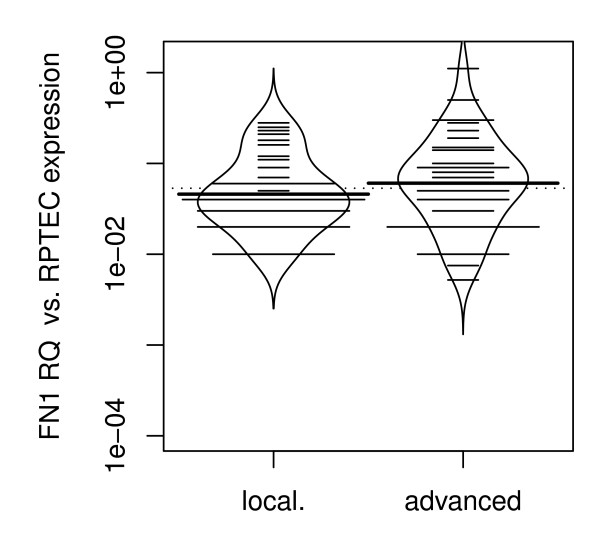
**Bean plot analysis of sage specific Expression in clear cell RCC: no significant difference in FN1 mRNA expression between organ-confined (pT ≤ 2 and N0/M0) and advanced (pT≥3 and/or N+/M+) RCC**. Abbreviations: FN1RQ = *Relative quantity of FN1 mRNA expression analysis using RPTEC primary cells as a biological calibrator; *local = *organ-confined RCC; *advanced = *advanced RCC*.

Furthermore, we compared *FN1 *mRNA expression between patients with organ-confined RCC (pT ≤ 2 and N0/M0) and advanced disease (pT≥3 and/or N+/M+). Both, organ-confined and advanced disease showed significantly higher levels of *FN1 *mRNA expression compared to normal renal tissues (p = 0.022 and p < 1×10^-7 ^respectively; ANOVA; figure 3). Patients with advanced disease had higher *FN1 *expression when compared to organ-confined disease (p < 0.001; Wilcoxon rank sum test; figure 3). We then divided the cohort in two groups, clear cell RCC and papillary RCC. Interestingly, when applying subgroup analysis we found a significantly higher *FN1 *mRNA expression between organ-confined and advanced disease in the papillary RCC and not in the clear cell RCC group (p = 0.02 vs. p = 0.2; Wilcoxon rank sum test, figures 4 and 5).

## Discussion

*FN1 *mRNA expression and its relationship to clinicopathological parameters in RCC has not yet been investigated. Functional studies have shown that *FN *and its receptors are important in mediating cell adhesion, migration, signal transduction and possibly in prevention of apoptosis [18]. It is a component of the extracellular matrix, and cells adhere to *FN *via the integrin transmembrane receptors. Either up-regulation or down-regulation [19-21] of *FN *has been reported in several human cancers. The ability to promote epithelial cell migration and suppression of apoptosis, suggest that *FN *overexpression promote various tumors types [22,23].

In the present study, a significantly increased expression of *FN1 *mRNA in RCC compared to normal renal tissue and oncocytoma has been shown. Both organ-confined (pT ≤ 2 and N0/M0) and advanced disease (pT≥3 and/or N+/M+) showed significant higher levels of *FN1 *mRNA expression compared to normal renal tissue. Moreover, patients with advanced disease had higher *FN1 *expression when compared to organ-confined disease. This supports the hypothesis that *FN *contributes to renal carcinogenesis and/or RCC progression.

There have been previous functional studies suggesting *FN *as a candidate marker for aggressive RCC. The first studies focusing on different RCC cell lines showed altered *FN *secretion and the possibility to influence mobility and invasiveness of these malignant cells [24-26].

A further interesting finding in our study was a significant increase in *FN1 *mRNA expression in clear cell RCC compared to papillary RCC. Our results generally suggest that an increased *FN1 *expression is correlated to a more progressive disease in RCC. Moreover, higher *FN1 *expression in clear cell RCC as compared to papillary RCC support the common belief that papillary tumors are less aggressive [27]. On the other hand current data indicates that once papillary RCC has spread, it is associated with a poor survival and a higher risk of resistance to systemic therapy compared to clear cell RCC [28]. This statement is supported by the fact that we found a significant higher *FN1 *mRNA expression between organ-confined and advanced disease in papillary RCC but not for clear cell RCC. Papillary RCC can be sub classified into types 1 and 2 as well as into less and more aggressive tumors. *FN1 *might be used to differentiate between papillary RCC with less or more aggressive potential.

## Conclusion

To our knowledge, this is the first study to show that *FN1 *mRNA expression is significantly higher in RCC compared to normal renal tissue and oncocytoma. *FN1 *mRNA expression might serve as a marker for RCC aggressiveness, indicating early systemic progression particularly for patients with papillary RCC. Therefore, our data on *FN1 *encourages further investigations to enlighten the future role of *FN1 *in RCC.

## Pre-publication history

The pre-publication history for this paper can be accessed here:

http://www.biomedcentral.com/1471-2407/10/503/prepub
